# *β*-Cyclodextrin Production by Cyclodextrin Glucanotransferase from an Alkaliphile* Microbacterium terrae* KNR 9 Using Different Starch Substrates

**DOI:** 10.1155/2016/2034359

**Published:** 2016-08-25

**Authors:** Kiransinh N. Rajput, Kamlesh C. Patel, Ujjval B. Trivedi

**Affiliations:** ^1^Department of Microbiology and Biotechnology, University School of Sciences, Gujarat University, Navrangpura, Ahmedabad, Gujarat 380 009, India; ^2^Department of Microbiology, BRD School of Biosciences, Sardar Patel Maidan, Sardar Patel University, Satellite Campus, Bakrol, Vallabh Vidyanagar, Gujarat 388 120, India

## Abstract

Cyclodextrin glucanotransferase (CGTase, EC 2.4.1.19) is an important member of *α*-amylase family which can degrade the starch and produce cyclodextrins (CDs) as a result of intramolecular transglycosylation (cyclization). *β*-Cyclodextrin production was carried out using the purified CGTase enzyme from an alkaliphile* Microbacterium terrae* KNR 9 with different starches in raw as well as gelatinized form. Cyclodextrin production was confirmed using thin layer chromatography. Six different starch substrates, namely, soluble starch, potato starch, sago starch, corn starch, corn flour, and rice flour, were tested for CD production. Raw potato starch granules were found to be the best substrate giving 13.46 gm/L of cyclodextrins after 1 h of incubation at 60°C. Raw sago starch gave 12.96 gm/L of cyclodextrins as the second best substrate. To achieve the maximum cyclodextrin production, statistical optimization using Central Composite Design (CCD) was carried out with three parameters, namely, potato starch concentration, CGTase enzyme concentration, and incubation temperature. Cyclodextrin production of 28.22 (gm/L) was achieved with the optimized parameters suggested by the model which are CGTase 4.8 U/L, starch 150 gm/L, and temperature 55.6°C. The suggested optimized conditions showed about 15% increase in *β*-cyclodextrin production (28.22 gm/L) at 55.6°C as compared to 24.48 gm/L at 60°C. The degradation of raw potato starch granules by purified CGTase was also confirmed by microscopic observations.

## 1. Introduction

Cyclodextrins are cyclic oligosaccharides commonly composed of six, seven, or eight D-glucose units (*α*-, *β*-, and *γ*-cyclodextrins, resp.) joined by *α*-(1, 4) glycosidic bonds. Cyclodextrin molecules have hydrophilic outside, which can dissolve in water, and hydrophobic nonpolar cavity, which is described as a “microheterogeneous environment” [1]. Because of this unique property, CDs can form molecular inclusion complexes (host-guest complexes) with a wide range of solid, liquid, and gaseous compounds and hence have found various applications in the field of medicine, food, pharmaceuticals, and cosmetics [2, 3].

Cyclodextrins (CDs) are produced as a result of intramolecular transglycosylation (cyclization) reaction during degradation of starch by CGTase enzyme. The CGTase is a multifunctional enzyme and catalyzes four different reactions: cyclization, disproportionation, coupling, and weak hydrolysis reaction [4].

Cyclodextrin production mainly depends on the process and incubation conditions used, while the distribution of *α*-, *β*-, and *γ*-CDs is highly dependent on the nature of the enzyme used [5]. Cyclodextrin production is reported from different starch sources like potato, corn, wheat, rice, and tapioca [6]. The major problem when starch is used as raw material for cyclodextrin production is the high viscosity of the reaction system which impedes stirring and contact between the enzyme and substrate. Therefore, a preliminary treatment of starch by physical, chemical, and enzymatic methods has been recommended [7, 8]. During gelatinization, the crystalline structure of starch is disrupted by heating in presence of water. Gelatinized starch swells irreversibly creating larger surface/volume ratio for enzymatic reaction [9–11].

## 2. Materials and Methods

### 2.1. Materials

*β*-Cyclodextrin was purchased from HiMedia, Mumbai, India. Soluble starch, yeast extract, and peptone were obtained from Qualigens, India. Phenolphthalein was purchased from Merck India Ltd. Finely ground corn flour and rice flour were collected from local flour mills and were sieved through fine cheese cloth to get fine powder. All other chemicals used were of analytical grade.

### 2.2. Organism, CGTase Production, and Purification

The CGTase producing organism used in this study was isolated from native soil in our laboratory as described by Park et al. [12]. This natural bacterial isolate was identified and deposited as* Microbacterium terrae* MTCC 8083 at IMTECH, Chandigarh, India [13].

CGTase production was carried out using 100 mL medium containing 20 g/L soluble starch, 10 g/L yeast extract, 1.0 g/L K_2_HPO_4_, 0.2 g/L MgSO_4_·7H_2_O, and 10 g/L Na_2_CO_3_ (autoclaved separately) in 250 mL flasks at 30°C, 150 rpm, on rotary shaker for 72 h. After incubation, cells were removed by centrifugation and supernatant was used for enzyme purification.

Cyclodextrin glucanotransferase enzyme was purified by starch adsorption method described by Martins and Hatti-Kaul [14] with slight modifications. In crude enzyme, ammonium sulphate was added to a concentration of 20% (w/v) saturation and kept for 2 h at 4°C on stirrer followed by centrifugation at 9000 ×g, 4°C for 20 min. After centrifugation, the supernatant was carefully transferred to another flask discarding the pellet of protein. To this supernatant, 5% (w/v) corn starch was added and kept for 1 h at 8°C with constant moderate stirring to allow CGTase enzyme adsorption. The mixture was then centrifuged at 5000 rpm for 10 min and the settled pellet of starch with adsorbed CGTase and other proteins was washed twice with 10 mL of cold distilled water to remove other unbounded proteins. The adsorbed CGTase was eluted from the corn starch by incubating it with 5 mL of 1 mM *β*-CD in 50 mM phosphate buffer, pH 6.0, for 30 min at 37°C with stirring. After that, it was centrifuged at 9000 ×g, 4°C for 20 min, and after centrifugation desorbed CGTase containing supernatant was carefully transferred to another tube. The elution was repeated once again with 2.0 mL of the elution buffer. The eluted fractions were pooled together and dialyzed against 50 mM Na-phosphate buffer, pH 6.0, at 8°C for 24 h with three buffer changes.

### 2.3. *β*-Cyclodextrin Estimation

CGTase activity and *β*-cyclodextrin production were determined by phenolphthalein assay method described by Goel and Nene [15] with minor modification. 100 *μ*L of appropriately diluted purified enzyme was incubated with 1.0 mL of 50 mg soluble starch in sodium phosphate buffer (50 mM, pH 6.0) at 60°C for 30 min. The reaction was stopped by quickly cooling the tubes on ice. Four milliliters of working phenolphthalein solution was added, the tubes were vortexed, and the absorbance of the mixture was immediately measured at 550 nm. The working phenolphthalein solution was prepared by adding 1 mL of phenolphthalein stock (4 mM in ethanol) to 100 mL of 125 mM Na_2_CO_3_ prepared in 4% ethanol. The standard *β*-cyclodextrin estimation was also carried out using the same method. One enzyme unit is defined as the amount of enzyme that produced one *μ*mole of *β*-cyclodextrin per minute under assay conditions.

### 2.4. Cyclodextrin Production Using Different Gelatinized Starches

Different starch substrates were tested for *β*-cyclodextrin production using appropriately diluted purified enzyme. Six different starch substrates, namely, soluble starch, potato starch, sago starch, corn starch, corn flour, and rice flour, were tested for *β*-CD production. 10 mL of each starch (20 gm/L) in 50 mM phosphate buffer (pH 6.0) was heated in boiling water bath (10 min) for gelatinization. After cooling down to 60°C, 100 *μ*L of appropriately diluted enzyme (4.8 U/L) was added in each flask and incubated at 60°C for *β*-cyclodextrin production. After 1 h and 2 h of incubation period, samples were withdrawn and *β*-CD production was estimated by phenolphthalein method as described above.

### 2.5. Cyclodextrin Production Using Different Raw Starches

Preliminary experiments of different raw starch degradation using purified CGTase were successful. In subsequent set of experiments, three starch substrates, namely, potato starch, corn starch, and sago starch, were tested in raw form for *β*-CD production with double CGTase concentration. A soluble starch was also kept as control for *β*-CD production. Reaction mixture of each starch (100 gm/L) in 10 mL phosphate buffer (50 mM, pH 6.0) and 100 *μ*L of appropriately diluted enzyme (9.6 U/L) were prepared and incubated at 60°C for *β*-CD production. Samples withdrawn after 1 h and 2 h were centrifuged to remove the suspended raw starch particles and then supernatants were analysed for *β*-cyclodextrin production by phenolphthalein method.

### 2.6. Optimization of *β*-Cyclodextrin Production Using Central Composite Design (CCD)

Enzymatic production of cyclodextrin depends on the reaction conditions like pH, temperature, concentration of substrate, concentration of enzyme, and incubation time. In this study, we have selected CGTase concentration (Units/L), potato starch concentration (gm/L), and temperature (°C) as the independent variables. Central Composite Design (CCD) was generated using the Design-Expert software (Stat-Ease Inc., Minneapolis, MN, USA, version 7.0.4) to test the statistical significance of these variables on *β*-cyclodextrin production. Their coded levels and actual values are shown in Table 1.

For statistical calculations, the relation between the coded values and actual values was calculated according to the following:

(1) $$\begin{array}{c} X_{i}=\frac{A_{i}-A_{0}}{\Delta A}, \end{array}$$

where *X*_*i*_ is the coded value of the variable, *A*_*i*_ is the actual value, *A*_0_ is the actual value of *A*_*i*_ at the centre point, and Δ*A* is the step change.

A CCD with six axial points and six replications at the centre points leading to a total number of 20 experiments was employed for the optimization of *β*-cyclodextrin production (Table 2). The second-order polynomials were calculated using the statistical package to estimate the response of the independent variable and develop a mathematical model.

(2) Y=β0+β1A+β2B+β3C+β12AB+β13AC+β23BC+β11A2+β22B2+β33C2,

where *Y* is the predicted response; *A*, *B*, and *C* are the independent variables; *β*_0_ is the offset term; *β*_1_, *β*_2_, and *β*_3_ are the linear coefficients; *β*_12_, *β*_13_, and *β*_23_ are the interaction coefficients; and *β*_11_, *β*_22_, and *β*_33_ quadratic coefficients.

Different concentrations of raw potato starch were taken in each tube containing 10 mL phosphate buffer (50 mM, pH 6.0). Appropriately diluted purified enzyme was added to achieve required enzyme units in each experimental run as shown in Table 2. The enzyme-substrate systems were then incubated at respective temperature in a shaking water bath for 2 h. After incubation, *β*-cyclodextrin production was measured using the phenolphthalein method.

### 2.7. Detection of Cyclodextrin Production by TLC

Presence of *β*-cyclodextrin in gelatinized soluble starch with purified CGTase was detected using thin layer chromatography (Silica gel 60, Merck India Ltd.). Aliquot from the abovementioned test was spotted on the TLC plate along with glucose, standard *β*-cyclodextrin, and gelatinized soluble starch (without enzyme) as control. The plate was developed with a solvent system containing dioxane and 25% ammonia solution (1 : 1 v/v). The *β*-cyclodextrins produced were detected by spraying the plate with 50% ethanolic sulphuric acid solution followed by heating at 100°C for 10–15 min.

### 2.8. Microscopic Examination of Degraded Raw Potato Starch

As purified CGTase is able to degrade raw potato starch and produce cyclodextrins, we examined the degradation of these raw potato starch granules in optical microscope (Carl Zeiss, Germany; Image Analyser KS300, 3.0).

## 3. Results and Discussion

### 3.1. CGTase Production and Purification

*Microbacterium terrae* KNR 9 produced 0.9 U/mg of crude CGTase enzyme at 30°C, 150 rpm, on rotary shaker after 72 h. After incubation, cells were removed by centrifugation and supernatant was processed for enzyme purification. CGTase from* Mic. terrae* KNR 9 could be purified to homogeneity in a single step purification using starch adsorption method described earlier with 45.22 U/mg of specific activity, 50-fold purification, and 33% purification yield.

### 3.2. *β*-Cyclodextrin Production Using Different Gelatinized Starch Substrates

Among the different gelatinized starch sources used for *β*-CD production, sago, potato, and corn starch showed higher *β*-cyclodextrin production while the rest of the starch sources tested showed comparatively lower *β*-CD production (Table 3). It is well known that CGTase prefers amylopectin as starch source for CD production over amylose [7]. The tapioca starch was the best source for CDs production using CGTase from* Bacillus* sp. G1 [6]. Gawande and Patkar [16] found soluble starch as the best substrate for CGTase from* Klebsiella pneumoniae* AS-22.

### 3.3. *β*-Cyclodextrin Production Using Different Raw Starches

In subsequent experiments, corn, sago, and potato starches were checked for *β*-CD production for 1 h and 2 h incubation period. Among the tested starch substrates, potato, sago, and even soluble starch showed better *β*-CD production in raw form than the gelatinized ones. The highest *β*-CD production was obtained with potato starch (Table 4). Kim et al. [17] have demonstrated that structure of starches may be partially destroyed above 75°C upon gelatinization which might not be favorable for *β*-CD production. However, very low *β*-CD production observed with raw corn starch indicates that it requires a prior heat treatment for better *β*-CD production as shown in Table 3. Goel and Nene [15] reported the raw starch degradation by* B. firmus* CGTase and maximum CD production with tapioca starch.Pishtiyski and Zhekova [18] reported that* B. megaterium* CGTase prefers high molecular mass starches and requires *α*-amylase treatment for cyclodextrin production. So, further optimization of *β*-CD production was done using raw potato starch without gelatinization.

### 3.4. Optimization of *β*-Cyclodextrin Production Using Central Composite Design (CCD)

Different combinations of substrate and enzyme concentrations were tested at a temperature range of 55–65°C with the help of CCD. Results of *β*-cyclodextrin production obtained after 2 h of incubation with different potato starch substrate concentration, CGTase concentration, and temperature are shown in Table 5. On the basis of results, analysis of variance was carried out using Fisher's test to determine the statistical significance of the selected variables. The regression equation obtained after analysis of variance gives the production of cyclodextrin using purified CGTase as a function of enzyme-substrate (potato starch) concentration and temperature.

(3) CGTase U/mL=−903.172−10.1864×CGTase+1.43584×potato  starch+29.47413×temperature−0.00868×CGTase×potato  starch+0.175313×CGTase×temperature−0.02389×potato  starch×temperature+0.089902×CGTase2+0.000568×potato  starch2−0.23653×temperature2.

From the ANOVA analysis, model *F*-value of 30.68 showed very high confidence level with the corresponding *p* value of <0.0001. The coefficient of determination, *R* ^2^, of the model is 0.9650 which indicates that the model is able to explain 96.50% of the data variability; only 3.5% of the total variation is not explained (Table 6). The predicted *R* ^2^ value of the model is 0.7717 which is in reasonable agreement with the adjusted *R* ^2^ of 0.9336. The *p* value of each variable in terms of linear interaction and squared effects is shown in Table 6. In this set of experiments, linear effect of starch and temperature, interaction effects of enzyme** ×** temperature and starch** ×** temperature, and squared terms of starch and temperature were found significant. Test of goodness of fit indicates that *p* value for the term “lack of fit” is 0.1193, which is larger than 0.05 indicating that “lack of fit” of the model is “not significant” which is required. In other words, the regression model is fit with the response obtained in the experimental runs and it can be used in predicting the response within the tested range of variables.

**Figure 1:**
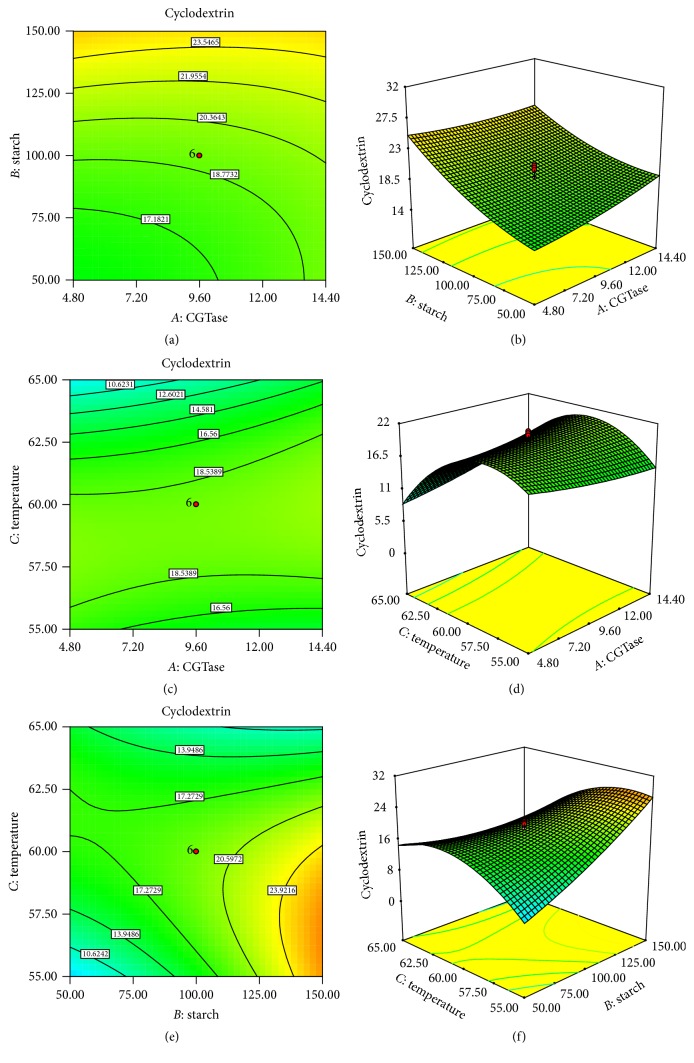
Contour and response surface plots showing interaction effects of variables on *β*-cyclodextrin production.

Response surface plots and 2D contour plots were generated showing the effect of two independent variables on *β*-cyclodextrin production keeping the other at its central level (Figure 1). Interaction effect of enzyme CGTase and starch concentration shown in Figures 1(a) and 1(b) indicates that increase in potato starch concentration has favorable impact on *β*-CD production but increase in CGTase concentration does not show any positive effect. Figures 1(c) and 1(d) show the CD production as an interaction of CGTase concentration and temperature keeping the starch at its central level. Here, central range of temperature is found optimum but CGTase concentration has no impact on *β*-CD production. As shown in Figures 1(e) and 1(f), potato starch concentration as substrate has profound effect on CD production and its higher concentration is favorable irrespective of temperature. At higher temperatures like 65°C, the CD production decreased, probably due to denaturation of enzyme. Consequently, lower range of temperatures and higher starch concentration have shown very good CD production and hence their interaction effect was found significant.

The optimized parameters suggested by the model are CGTase, 4.8 (U/L), starch 150 gm/L, and temperature 55.6°C with the predicted response 29.67 gm/L of *β*-CD production. An experimental run was kept with the conditions suggested by the model and 28.22 gm/L of *β*-CD production was obtained which is comparable to other reports. Goel and Nene [15] obtained about 16 gm/L cyclodextrin production with tapioca starch. Cyclodextrin production of about 22 gm/L has been reported using raw corn starch without pretreatment [17]. Pishtiyski and Zhekova [18] achieved up to 16 gm/L *β*-CD production with different starch substrates. Rauf et al. [19] have reported maximum 8.43 gm/L of cyclodextrin production from ungelatinized sago starch using statistical methods.

### 3.5. Detection of *β*-Cyclodextrin Production by Thin Layer Chromatography

**Figure 2:**
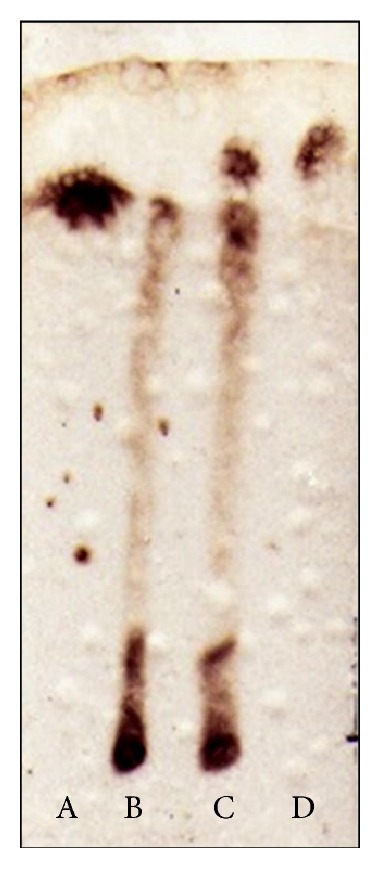
Detection of cyclodextrin production by TLC. Lane, A-glucose; lane, B-gelatinized starch (control); lane, C-gelatinized starch with enzyme; lane, D-standard *β*-cyclodextrin.

The *β*-cyclodextrin production was confirmed on TLC and detectable amount of *β*-cyclodextrin was produced from gelatinized soluble starch at 60°C in 1 h (lane C) which can be compared with *β*-cyclodextrin standard (lane D) on TLC plate (Figure 2). In the enzymatic reaction (lane C), the other spot that appeared may be of glucose, as its migration is comparable with standard glucose (lane A). The presence of glucose might have been observed because of inherent presence of glucose in the soluble starch and not because of enzymatic degradation as gelatinized soluble starch control (without enzyme, lane B) also showed the presence of glucose. Here, purified CGTase has shown the cyclization as its main activity and negligible hydrolysis.

### 3.6. Microscopic Examination of Potato Starch for Its Degradation

**Figure 3:**
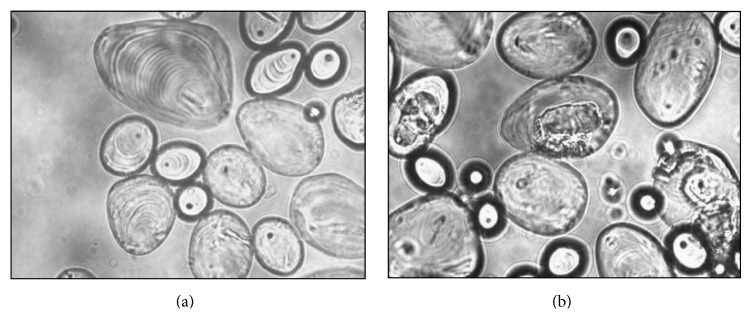
Raw potato starch degradation observed under light microscope ((a) control, (b) enzymatic degradation).

As higher *β*-CD production was obtained using potato starch granules in raw form, microscopic observation of raw potato starch granules after degradation was carried out (Figure 3). Confirming the activity of the enzyme, many potato starch granules were observed in a degradation stage as shown in Figure 3(b) as compared to Figure 3(a) that is control. Another noticeable feature was that majority of the granules were primarily attacked on nuclear portion of the potato starch granules. Yamamoto et al. [20] have also reported the degradation of intact potato starch granules.

## 4. Conclusion

Cyclodextrin glucanotransferase produced by* Microbacterium terrae* KNR 9 can degrade gelatinized form as well as the raw form of all the different starch substrates tested except corn starch. Raw potato starch and sago starch were found as the most suitable raw starches for this enzyme. Thus, purified CGTase has potential for use in enzymatic production of *β*-cyclodextrins.

## Figures and Tables

**Table 1:** Independent variables and their levels studied in CCD.

Variables	Coded levels				
	−1.682	−1	0	1	1.682
*A*: CGTase (units/L)	1.52	4.8	9.6	14.8	17.86
*B*: potato starch (gm/L)	15.91	50	100	150	184.09
*C*: temperature (°C)	51.59	55	60	65	68.41

**Table 2:** The CCD used for *β*-cyclodextrin production with its coded values.

Standard run number	CGTase (units/L)	Potato starch (gm/L)	Temperature (°C)
1	−1	−1	−1
2	1	−1	−1
3	−1	1	−1
4	1	1	−1
5	−1	−1	1
6	1	−1	1
7	−1	1	1
8	1	1	1
9	−1.682	0	0
10	1.682	0	0
11	0	−1.682	0
12	0	1.682	0
13	0	0	−1.682
14	0	0	1.682
15	0	0	0
16	0	0	0
17	0	0	0
18	0	0	0
19	0	0	0
20	0	0	0

**Table 3:** *β*-CD production from different gelatinized starches.

Starch source	*β*-Cyclodextrin production (gm/L)	
	1 h	2 h
Soluble starch	1.84	1.77
Potato starch	4.19	8.43
Corn starch	3.73	7.89
Sago starch	4.22	8.91
Rice flour	3.18	4.96
Corn flour	1.36	4.42

**Table 4:** *β*-CD production from different raw starches.

Starch source	*β*-Cyclodextrin production (gm/L)	
	1 h	2 h
Corn starch	0.170	0.272
Sago starch	12.92	22.78
Potato starch	13.46	24.48
Soluble starch	11.70	19.04

**Table 5:** Actual and predicted *β*-cyclodextrin production in CCD.

Std.	CGTase (Units/L)	Starch (gm/L)	Temp. (°C)	*β*-Cyclodextrin (gm/L)	
				Actual	Predicted
1	4.8	50	55	10.54	8.09
2	14.4	50	55	07.82	7.54
3	4.8	150	55	27.88	29.58
4	14.4	150	55	25.84	24.86
5	4.8	50	65	10.20	11.26
6	14.4	50	65	20.74	19.12
7	4.8	150	65	8.50	8.86
8	14.4	150	65	10.03	12.56
9	1.52	100	60	19.72	19.35
10	17.86	100	60	21.76	22.00
11	9.6	15.91	60	14.96	16.95
12	9.6	184.09	60	31.62	29.50
13	9.6	100	51.6	05.10	6.32
14	9.6	100	68.4	00.0	−1.35
15	9.6	100	60	18.36	19.21
16	9.6	100	60	20.74	19.21
17	9.6	100	60	18.70	19.21
18	9.6	100	60	20.40	19.21
19	9.6	100	60	17.00	19.21
20	9.6	100	60	20.06	19.21

**Table 6:** ANOVA regression analysis for CGTase production using CCD.

Source	Sum of squares	df	Mean square	*F*-Value	*p* value Prob > *F*
Model	1174.25	9	130.47	30.68	<0.0001
*A*: CGTase	8.44	1	8.44	1.98	0.1890
*B*: potato starch	190.21	1	190.21	44.73	<0.0001
*C*: temperature	71.21	1	71.21	16.75	0.0022
*AB*	8.67	1	8.67	2.04	0.1837
*AC*	35.40	1	35.40	8.32	0.0162
*BC*	285.24	1	285.24	67.09	<0.0001
*A* ^2^	3.86	1	3.86	0.90	0.3629
*B* ^2^	29.03	1	29.03	6.82	0.0259
*C* ^2^	503.92	1	503.92	118.52	<0.0001

## References

[1] Szejtli J. (1998). Introduction and general overview of cyclodextrin chemistry. *Chemical Reviews*.

[2] Horikoshi K. (1979). Production and industrial applications of *β*-cyclodextrins. *Process Biochemisty*.

[3] Hedges A. R. (1998). Industrial applications of cyclodextrins. *Chemical Reviews*.

[4] van der Veen B. A., Van Alebeek G.-J. W. M., Uitdehaag J. C. M., Dijkstra B. W., Dijkhuizen L. (2000). The three transglycosylation reactions catalyzed by cyclodextrin glycosyltransferase from *Bacillus circulans* (strain 251) proceed via different kinetic mechanisms. *European Journal of Biochemistry*.

[5] Schmid G. (1989). Cyclodextrin glycosyltransferase production: yield enhancement by overexpression of cloned genes. *Trends in Biotechnology*.

[6] Sian H. K., Said M., Hassan O. (2005). Purification and characterization of cyclodextrin glucanotransferase from alkalophilic *Bacillus* sp. G1. *Process Biochemistry*.

[7] Biwer A., Antranikian G., Heinzle E. (2002). Enzymatic production of cyclodextrins. *Applied Microbiology and Biotechnology*.

[8] Charoenlap N., Dharmsthiti S., Sirisansaneeyakul S., Lertsiri S. (2004). Optimization of cyclodextrin production from sago starch. *Bioresource Technology*.

[9] Tester R. F., Karkalas J., Qi X. (2004). Starch—composition, fine structure and architecture. *Journal of Cereal Science*.

[10] Goh K. M., Mahadi N. M., Hassan O., Abdul Rahman R. N. Z. R., Illias R. M. (2007). The effects of reaction conditions on the production of *γ*-cyclodextrin from tapioca starch by using a novel recombinant engineered CGTase. *Journal of Molecular Catalysis B: Enzymatic*.

[11] Ratnayake W. S., Jackson D. S. (2007). A new insight into the gelatinization process of native starches. *Carbohydrate Polymers*.

[12] Park C. S., Park K. H., Kim S. H. (1989). A rapid screening method for alkaline .BETA.-cyclodextrin glucanotransferase using phenolphthalein-methyl orange-containing-solid medium. *Agricultural and Biological Chemistry*.

[13] Rajput K. N., Patel K. C., Trivedi U. B. (2016). Screening and selection of medium components for cyclodextrin glucanotransferase production by new alkaliphile *Microbacterium terrae* KNR9 using Plackett-Burman design. *Biotechnology Research International*.

[14] Martins R. F., Hatti-Kaul R. (2003). *Bacillus agaradhaerens* LS-3C cyclodextrin glycosyltransferase: activity and stability features. *Enzyme and Microbial Technology*.

[15] Goel A., Nene S. (1995). A novel cyclomaltodextrin glucanotransferase from *Bacillus firmus* that degrades raw starch. *Biotechnology Letters*.

[16] Gawande B. N., Patkar A. Y. (2001). Purification and properties of a novel raw starch degrading-cyclodextrin glycosyltransferase from *Klebsiella pneumoniae* AS-22. *Enzyme and Microbial Technology*.

[17] Kim T.-J., Kim B.-C., Lee H.-S. (1997). Production of cyclodextrin using raw corn starch without a pretreatment. *Enzyme and Microbial Technology*.

[18] Pishtiyski I., Zhekova B. (2006). Effect of different substrates and their preliminary treatment on cyclodextrin production. *World Journal of Microbiology and Biotechnology*.

[19] Rauf Z. A., Ilias R. M., Mahadi N. M., Hassan O. (2008). Experimental design to optimization of beta cyclodextrin production from ungelatinized sago starch. *European Food Research and Technology*.

[20] Yamamoto K., Zhang Z. Z., Kobayashi S. (2000). Cycloamylose (cyclodextrin) glucanotransferase degrades intact granules of potato raw starch. *Journal of Agricultural and Food Chemistry*.

